# Associations between neutrophil-lymphocyte ratio with all-cause mortality, major adverse vascular events and progression of diabetic kidney disease in type 2 diabetes mellitus

**DOI:** 10.3389/fendo.2025.1695139

**Published:** 2025-11-24

**Authors:** Ying Jie Chee, Xiaoe Doris Zhang, Rinkoo Dalan

**Affiliations:** 1 Department of Endocrinology, Tan Tock Seng Hospital, Singapore, Singapore; 2 Lee Kong Chian School of Medicine, Nanyang Technological University, Singapore, Singapore; 3 Clinical Research and Innovations Office, Tan Tock Seng Hospital, Singapore, Singapore

**Keywords:** diabetes mellitus type 2, neutrophil-lymphocyte (N/L ratio), mortality, major adverse vascular complications, renal outcomes

## Abstract

**Introduction:**

The neutrophil lymphocyte ratio (NLR) is a readily accessible marker of systemic inflammation. This study evaluated the association between NLR with all-cause mortality, major adverse vascular events and diabetic kidney disease (DKD) progression in a multiethnic cohort of adult type 2 diabetes mellitus (T2DM) individuals in Singapore.

**Methods:**

Demographic, anthropometric, biochemistry, mortality and major adverse vascular events (MAVE) were obtained from electronic medical records up to June 30, 2024. Composite renal outcomes were defined as one of the following: decline in eGFR ≥ 40%, decline in eGFR to ≤ 15ml/min/1.73m^2^ or initiated maintenance dialysis. Multivariate Cox regression analyses were performed to evaluate associations between NLR, all-cause mortality, MAVE and composite renal outcomes.

**Results:**

In this cohort of 959 adult participants with T2DM, there was a significant association between NLR with all-cause mortality, MAVE, baseline albuminuria, renal function and progression of DKD. During the median follow-up of 9.4 years, there were 367 (38.3%) mortalities, 222 (23.1%) cases of MAVE and 285 (30%) participants who developed a renal outcome. The highest NLR was associated with a 1.6-fold increased risk for all-cause mortality (HR 1.63; 95% 1.18 - 2.27, p=0.003), 2.7-fold increased risk of MAVE (HR 2.71; 95% CI 1.75 - 4.20; p<0.001) and 1.55 (HR 1.55, 95% CI 1.09 - 2.19, p=0.014) increased risk of having a renal event compared to the lowest NLR tertile after adjusting for confounders.

**Conclusion:**

Elevated NLR is independently associated with all-cause mortality, MAVE and composite renal outcomes in T2DM. NLR may be considered a potential clinical biomarker of adverse outcomes for use in routine care.

## Introduction

Type 2 diabetes mellitus (T2DM) affects more than 460 million people worldwide and vascular complications are the main cause of mortality and morbidity in these individuals (1, 2). Data from the DISCOVER global study revealed microvascular complications occurring in nearly 20% while macrovascular complications in 20% at baseline (3). At the end of 3 years of follow up, the burden of microvascular complications increased to nearly one-third of the study cohort, with progression of diabetic nephropathy occurred in about 16% while macrovascular complications taking place in about 17% (4).

Current cardiovascular outcome studies in T2DM focus on traditional 3-point or 4-point major adverse cardiovascular events (MACE), comprising of fatal, nonfatal acute myocardial infarction and stroke. However, focusing on MACE alone tends to overlook peripheral vascular disease (PVD), which can affect up to 50% of individuals with T2DM in developed countries and confer additional cardiovascular risks (5). Adverse cardiovascular events were found to occur in more than of individuals with T2DM and PVD over 7 years (6). Despite the high prevalence of PVD, it is not routinely assessed in cardiovascular outcome studies. The under recognition of PVD as an outcome measure in T2DM has led to a recent call for a more comprehensive approach to vascular complications assessment, one that includes PVD as an additional marker of surrogate cardiovascular outcome. To capture the global burden of vascular complications, the term major adverse vascular events (MAVE) was proposed to incorporate the concept of shared pathophysiology and involvement of multiple vascular territories in T2DM (7).

The full blood count is a routinely performed blood test that is inexpensive, readily accessible with relatively short processing time. Further analysis of the differential counts provides further insights into risk stratification for vascular complications. The neutrophil lymphocyte ratio (NLR) represents a marker of inflammation that integrates both innate (neutrophils) and adaptive (lymphocytes) pathways (8). Elevated neutrophil counts are associated with inflammatory activation, release of proinflammatory cytokines and prothrombotic mediators, while lymphopenia reflects T-cell dysregulation or T-cell apoptosis as a counter-regulatory response to stress (9).

Emerging evidence supports the role of NLR in predicting cardiovascular outcomes (10). A longitudinal analysis of over 3000 individuals with T2DM in the NHANES cohort found that an elevated NLR was associated with all-cause mortality (11). This finding was corroborated in a Chinese cohort of T2DM individuals, in which NLR was associated with increased cardiovascular events (12). Despite available evidence on NLR, there are still gaps to be addressed. First, validation studies from Southeast Asian populations are lacking, given potential genetic and environmental differences that may affect the prognostic performance of NLR. Second, previous studies have predominantly focused on traditional MACE endpoints, excluding PVD. Third, most studies have limited duration of follow-up and might not fully capture the sustained association between NLR and vascular complications over a longer duration. Fourth, most studies focused either on cardiovascular complications or progression of diabetic kidney disease (DKD) as isolated outcomes, but not on both outcomes in the same cohort, limiting the assessment of NLR as an integrated and comprehensive marker of macrovascular and microvascular complications in T2DM.

Given the current gaps, we conducted a prospective observational study in a multiethnic Southeast Asian cohort of individuals with T2DM, employing MAVE as the composite endpoint with nearly 10 years follow-up to evaluate the associations of NLR as a biomarker of vascular complications and progression of DKD in T2DM.

## Methods

### Study cohort

All participants with T2DM on follow up at the Department of Endocrinology clinic at Tan Tock Seng Hospital between January 2013 to December 2014 who had a full blood count performed in the outpatient setting were recruited into this study. This study followed a prospective cohort design. The study was approved by the National Healthcare Group Ethnics Domain (DSRB ref: 2015/00368). Data were collected from participants’ electronic medical records (EMR). The following data fields were collected: demographics including age, sex, ethnicity; anthropometrics including height, weight, blood pressure and biochemical including full blood count with differential counts, glycated hemoglobin (HbA1c), lipid profiles, serum creatinine, urinary albumin and creatinine to derive the urinary albumin to creatinine ratio (uACR).

The biochemical data were obtained from blood samples performed as per participants’ routine laboratory tests prior to their outpatient clinic consultations. The neutrophil lymphocyte ratio (NLR) was determined by dividing the absolute neutrophil count by the absolute lymphocyte count.

### Outcome variables

The outcomes of interest include all-cause mortality, major adverse vascular events and composite renal outcomes. All-cause mortality data were retrieved from the participants’ EMRs. Follow-up data was not available if the participant was lost to follow-up or care was transferred to another hospital or primary care. Major adverse vascular events (MAVE) were defined as a composite of 4-point major adverse cardiovascular events (cardiovascular mortality, nonfatal myocardial infarction or stroke, unstable angina), peripheral vascular disease (PVD) (7) and associated complications using ICD-10-CM codes (13). The corresponding ICD codes for major adverse cardiovascular events included I20-25, representing the diagnoses of ischemic heart disease, acute myocardial infarction, angina; I63–66 and G46, which corresponded to the diagnoses of cerebral infarction; I70.21 to I70.26 and E11.5 to E11.6, indicating the presence of intermittent claudication or rest pain or ulceration or gangrene associated with atherosclerosis of peripheral arteries, in conjunction with T2DM diagnosis (13). To minimize misclassification of PVD associated complications, the clinical notes were reviewed to ensure the gangrene diagnosis was vascular, non-infectious in origin, and ulcers were ischemic and not purely neuropathic. We also considered other cardiovascular outcomes including type 2 myocardial infarction (I21.A1) and transient ischemic attack (G45.9). The estimated glomerular filtration rate (eGFR) was calculated using the CKD-EPI equation (14). Composite renal outcomes were defined as fulfilling one of the following criteria: decline in eGFR by 40% from baseline, eGFR reaching 15 ml/min/1.73m^2^ and below or initiation of dialysis (15). Albuminuria was defined per KDIGO guidelines (16). Microalbuminuria was defined as uACr 3.5 - 30mg/mmol; macroalbuminuria uACr 30 - 300mg/mmol and overt albuminuria as > 300mg/mmol. Progression of albuminuria is considered if there is a transition between less severe to more severe albuminuria category, for example, from no albuminuria to albuminuria or microalbuminuria to macroalbuminuria or worsening of pre-existing macroalbuminuria.

### Statistical analysis

NLR was analyzed as a continuous variable and categorized into tertiles: tertile 1 (≤ 1.88), tertile 2 (1.88-2.73), tertile 3 (≥ 2.74), corresponding to low, middle and high NLR categories, with the same cutoffs applied across all outcomes. Continuous variables were expressed as means and standard deviations (SD) or medians and interquartile ranges (IQR) depending on the distribution. Categorical variables were presented as frequencies and percentages. Non-parametric tests (Wilcoxon rank-sum and Kruskal-Wallis tests) were performed to assess baseline NLR with pre-existing coronary artery disease (CAD), albuminuria and renal function respectively. The Cox proportional hazard model was used to estimate the hazard ratios of NLR on the risk of outcomes (all-cause mortality, MAVE and composite renal outcomes). Three models were used to explore the associations. Model 1 was adjusted for age, sex and ethnicity. Model 2 was adjusted for model 1 plus body mass index (BMI). Model 3 was adjusted for model 2 plus SBP, HbA1c, total cholesterol, LDL-cholesterol, non-HDL cholesterol, baseline eGFR, pre-existing CAD or PVD, and the use of medications including angiotensin-converting enzyme (ACE) inhibitors or angiotensinogen receptor blockers (ARB) and statin. For composite renal outcomes, we adjusted for model 2 plus SBP, HbA1c, total cholesterol, LDL-cholesterol, non-HDL cholesterol, baseline eGFR, baseline uACR, pre-existing CAD or PVD, and the use of ACE inhibitors or ARB. The probability of survival outcomes was calculated according to the Kaplan-Meier method and compared using the log-rank test. The interactions between NLR tertiles with age, sex and ethnicity. Results were considered significant if p-value < 0.05. All statistical analyses were performed using R version 4.4.1.

## Results

In this cohort of 959 participants with T2DM, participants with higher NLR tertiles were older (p<0.001) with a greater proportion being males (p=0.002). The majority of participants with the highest NLR tertile were Chinese (78%) followed by Malays (14%) and Indians (8.5%) and other ethnic groups (4.7%). A greater proportion of participants with pre-existing CAD or PVD had the highest NLR tertile. The systolic blood pressure (SBP) was higher with each NLR tertile. There were no differences in the other variables including BMI, HbA1c, LDL cholesterol, non-HDL cholesterol among NLR tertiles. Regarding baseline eGFR and albuminuria, there was a greater proportion of participants with higher NLR tertiles having worse renal function and albuminuria. In terms of vascular complications, 26% of the study cohort had pre-existing coronary artery disease and 16% had PVD at the time when NLR was measured with a greater proportion of participants having higher NLR tertiles. The baseline characteristics of the study participants are presented in **Table 1**.

**Table 1:** Baseline characteristics of participants by NLR tertiles.

Characteristic	T1 (lowest) N = 320	T2 N = 319	T3 (highest) N = 320	Overall N = 959	p-value^2^
Age (years)	59 ± 13	61 ± 13	63 ± 12	61 ± 13	<0.001
Sex					0.002
Male	133 (42%)	163 (51%)	176 (55%)	472 (49%)	
Female	187 (58%)	156 (49%)	144 (45%)	487 (51%)	
Ethnicity					0.003
Chinese	197 (66%)	216 (71%)	237 (78%)	650 (68%)	
Malay	49 (16%)	35 (11%)	42 (14%)	126 (13%)	
Indian	51 (17%)	54 (18%)	26 (8.5%)	131 (13%)	
Others	23 (7.2%)	14 (4.4%)	15 (4.7%)	52 (5.4%)	
Pre-existing CAD	58 (18%)	89 (28%)	102 (32%)	249 (26%)	<0.001
Pre-existing PVD	36 (11%)	49 (15%)	71 (22%)	156 (16%)	<0.001
BMI (kg/m²)	25.4 (22.8, 29.9)	26.6 (23.4, 30.2)	26.7 (23.0, 30.7)	26.3 (23.1, 30.4)	0.3
SBP (mmHg)	130 (120, 146)	135 (124, 151)	140 (125, 155)	135 (122, 151)	0.002
DBP (mmHg)	71 (65, 78)	73 (66, 80)	72 (63, 80)	72 (65, 80)	0.2
HbA1c (%)	7.8 (6.8, 9.4)	7.80 (6.8, 9.2)	7.60 (6.8, 9.1)	7.70 (6.8, 9.2)	0.7
Total cholesterol (mmol/L)	4.6 (4.0, 5.4)	4.4 (3.7, 5.0)	4.3 (3.7, 5.1)	4.4 (3.8, 5.2)	0.005
LDL cholesterol (mmol/L)	2.5 (2.0, 3.0)	2.4 (1.9, 2.9)	2.4 (1.9, 3.0)	2.4 (2.0, 3.0)	0.2
Non-HDL cholesterol (mmol/L)	3.2 (2.7, 4.1)	3.1 (2.6, 3.8)	3.1 (2.6, 3.9)	3.2 (2.6, 3.9)	0.053
WBC (×10^9^/L)	7.0 (5.8, 8.2)	7.5 (6.2, 9.0)	8.4 (7.2, 9.9)	7.6 (6.3, 9.1)	<0.001
Absolute neutrophil (×10^9^/L)	3.6 (3.0, 4.2)	4.6 (3.7, 5.5)	5.9 (5.0, 7.1)	4.6 (3.6, 5.8)	<0.001
Absolute lymphocyte (×10^9^/L)	2.5 (2.1, 3.1)	2.1 (1.7, 2.4)	1.6 (1.3, 1.8)	2.0 (1.6, 2.5)	<0.001
eGFR category					<0.001
<15	6 (1.9%)	8 (2.5%)	33 (10%)	47 (4.9%)	
15–29.9	17 (5.3%)	34 (11%)	70 (22%)	121 (13%)	
30–59.9	67 (21%)	93 (29%)	77 (24%)	237 (25%)	
≥60	230 (72%)	184 (58%)	140 (44%)	554 (58%)	
Albuminuria category					<0.001
Normoalbuminuria (≤30)	215 (78%)	182 (68%)	141 (54%)	538 (67%)	
Microalbuminuria (>30–300)	54 (19%)	77 (29%)	99 (38%)	230 (29%)	
Macroalbuminuria (>300)	8 (2.9%)	7 (2.6%)	20 (7.7%)	35 (4.4%)	

Results were presented as mean ± SD; n (%); median (Q1, Q3) as appropriate.

### NLR and all-cause mortality

Over a median follow-up of 9.4 years, there were 367 mortalities. The most common cause of mortality was infection (16.6%), followed by coronary artery disease (12%) and malignancy (6.5%). Among the 61 infection-related mortalities, 46 (75.4%) were attributed to pneumonia and 15 were due to other infections, including urinary tract infection (n=5), hepatobiliary sepsis (n=3), bacteremia (n=2), infective endocarditis (n=2), gastroenteritis (n=1), uterine infection (n=1) and cellulitis (n=1). The mortality causes for this cohort are summarized in **Table 2a**.

**Table 2A:** All-cause mortality etiologies.

Outcome	Number (%) of events
All-cause mortality (n = 367)	
Infection	61 (16.6)
- Pneumonia	46 (75.4% of infection)
- Other infections (urinary tract infection, hepatobiliary sepsis, infective endocarditis, bacteremia, gastroenteritis, cellulitis)	15 (24.6% of infection)
Coronary artery disease	44 (12)
Cancer	24 (6.5)
Stroke	3 (0.8)
End-stage renal disease	11 (3)
Others	14 (3.8)
Unclear	210 (57.2)

**Figure 1:**
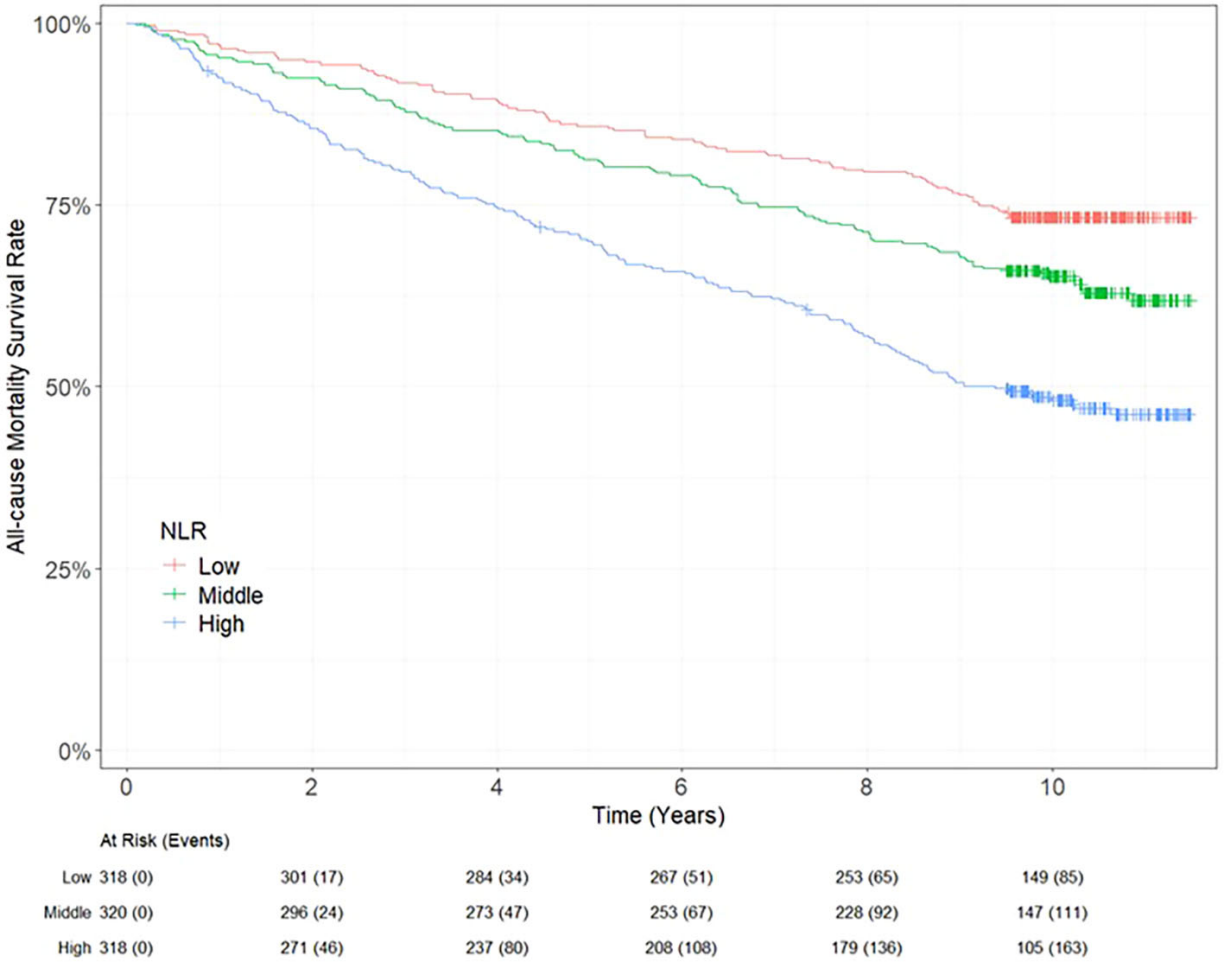
Kaplan Meier curves showing survival probability and the absolute number of at-risk T2DM participants with high, middle and low NLR tertiles.

A dose response relationship was observed between NLR tertile and all-cause mortality. In univariate Cox regression analysis, with the lowest NLR tertile as reference, the hazard ratio (HR) risk of all-cause mortality for middle and high NLR tertiles were 1.44 (95% CI: 1.09 - 1.90, p=0.01) and 2.41 (95% CI: 1.86 - 3.13, p<0.0001) respectively. In the final adjusted model for age, sex, ethnicity, BMI, SBP, HbA1c, lipid profiles, baseline eGFR, pre-existing CAD or PVD, use of angiotensin converting enzyme (ACE) inhibitor or angiotensin receptor blocker (ARB) and statin, the highest NLR was associated with a 1.63-fold increased risk for all-cause mortality (HR 1.63; 95% 1.18 – 2.27, p=0.003) (**Table 2b**). The Kaplan Meier survival curves showed that the cumulative probability for all-cause mortality differed for each tertile of NLR, with the lowest cumulative probability of 52% for the highest NLR tertile, followed by 65% for the middle NLR tertile and 74% for the lowest NLR tertile (**Figure 1**).

**Table 2B:** Cox regression analysis of association between NLR tertile and all-cause mortality.

NLR (tertile)	Unadjusted HR	p-value	Model 1 HR (95% CI)	p-value	Model 2 HR (95% CI)	p-value	Model 3 HR (95% CI)	p-value
Low	Reference							
Middle	1.44 (1.09 -1.90)	0.01	1.27 (0.95 - 1.68)	0.1	1.23 (0.91 - 1.67)	0.17	1.10 (0.79 - 1.55)	0.56
High	2.41 (1.86 - 3.13)	<0.001	2.09 (1.6 -2.72)	<0.001	2.02 (1.53 - 2.68)	<0.001	1.63 (1.18 - 2.27)	0.003

Model 1: adjusted for age, sex, ethnicity.

Model 2: adjusted for age, sex, ethnicity and BMI.

Model 3: adjusted for age, sex, ethnicity, BMI, SBP, HbA1c, total cholesterol, LDL, non-HDL, baseline eGFR, pre-existing CAD or PVD, use of ACE inhibitor or ARB and statin.

### NLR and MAVE

At baseline, the median NLR was higher in participants with pre-existing CAD (2.45 vs 2.16, p<0.001). During the 9.4 years of follow-up, majority of MAVE was nonfatal non-ST elevated myocardial infarction, which accounted for 40.5% of cases, followed by peripheral vascular disease (22.1%) and stroke or transient ischemic attack (15.8%). The etiologies of MAVE are summarized in **Table 3a**.

**Table 3A:** Summary of MAVE etiologies.

Outcome	Number (%) of events
MAVE (n = 222)	
Fatal AMI	18 (8.1)
Cardiac arrest	5 (2.3)
Sudden death	3 (1.4)
Nonfatal NSTEMI	90 (40.5)
Nonfatal STEMI	8 (3.6)
Type 2 MI	12 (5.4)
Unstable angina	2 (0.9)
Stroke	35 (15.8)
Peripheral vascular disease	49 (22.1)

In the unadjusted Cox regression analysis, high and middle NLR tertile was associated with a 3.9-fold (HR 3.89; 95% CI 2.7 - 5.61, p<0.001) and 1.9-fold (HR 1.89; 95% CI 1.27 - 2.80, p=0.001) increase in all-cause MAVE compared to the lowest NLR tertile. In the fully adjusted model, the middle NLR tertile was associated with a 1.59-fold increased risk of MAVE compared to the lowest NLR tertile (HR 1.59; 95% CI 0.99 - 2.52, p=0.05), while the highest NLR tertile was associated with a 2.71-fold increased risk of MAVE (HR 2.71; 95% CI 1.75 - 4.20; p<0.001) (**Table 3b**).

**Table 3B:** Cox regression analysis of association between NLR and MAVE.

NLR (tertile)	Unadjusted HR (95% CI)	p-value	Model 1 HR (95% CI)	p-value	Model 2 HR (95% CI)	p-value	Model 3 HR (95% CI)	p-value
Low	Reference							
Middle	1.89 (1.27 - 2.80)	0.001	1.86 (1.25 - 2.76)	0.002	1.79 (1.20 - 2.67)	0.005	1.59 (0.99 - 2.52)	0.05
High	3.89 (2.70 - 5.61)	<0.001	3.63 (2.51 - 5.25)	<0.001	3.43 (2.36 - 5.0)	<0.001	2.71 (1.75 – 4.20)	<0.001

Model 1: adjusted for age, sex, ethnicity.

Model 2: adjusted for age, sex, ethnicity and BMI.

Model 3: adjusted for age, sex, ethnicity, BMI, SBP, HbA1c, total cholesterol, LDL, non-HDL, baseline eGFR, pre-existing CAD or PVD, ACE-inhibitor or ARB and statin use.

### NLR and composite renal outcomes

The median time interval between the baseline and follow-up eGFR was 9 years with 10 patients having two eGFR readings with time intervals shorter than 1 year. The baseline NLR was associated with albuminuria and renal function in a step-wise manner. The median NLR progressively increased with declining renal function (2.05 vs 2.31 vs 2.86 vs 3.37 for eGFR ≥ 60 ml/min/1.73m^2^ vs eGFR 30–60 ml/min/1.73m^2^ vs eGFR 15–30 ml/min/1.73m^2^ vs eGFR < 15 ml/min/1.73m^2^, p<0.001). The median NLR progressively increased with worsening uACr (2.09 vs 2.46 vs 2.84 for normoalbuminuria vs microalbuminuria vs macroalbuminuria, p<0.001). Among 624 participants with follow-up uACr data, 288 (46.2%) remained normoalbuminuric; 107 (17.1%) progressed from normoalbuminuric to having albuminuria; 114 (18.3%) had worsening microalbuminuria while 115 (18.4%) had worsening macroalbuminuria. In terms of composite renal outcomes, among 904 participants with follow up renal function and who did not have end stage renal disease at recruitment, 285 (31.5%) participants sustained a renal event - 137 had eGFR declined by 40% or more, 25 had eGFR reaching 15 ml/min/1.73m^2^ not initiated dialysis, and 123 initiated maintenance dialysis (**Table 4a**). In the unadjusted analysis, compared to the lowest tertile of NLR, individuals in the middle tertile had a 1.58 increased risk (HR 1.58, 95% CI 1.17, 2.13, p=0.002), while those in the highest NLR tertile had a 2.12 increased risk of composite renal outcomes (HR 2.12, 95% CI 1.57, 2.86, p<0.001). In the fully adjusted model, the middle and highest NLR tertiles were associated with a 1.67-fold (HR 1.67, 95% CI 1.19, 2.33, p=0.003) and 1.55-fold increased risk (HR 1.55, 95% CI 1.09, 2.19, p=0.014) of experiencing a renal outcome (**Table 4b**).

**Table 4A:** Summary of composite renal outcomes.

Outcome	Number (%) of events
Composite renal outcomes (n = 285)	
eGFR declined ≥ 40%	137 (48.1)
eGFR ≤ 15	25 (8.8)
Initiated dialysis	123 (43.2)

**Table 4B:** Cox regression analysis of association between NLR and renal outcomes.

NLR (tertile)	Unadjusted HR (95% CI)	p-value	Model 1 HR (95% CI)	p-value	Model 2 HR (95% CI)	p-value	Model 3 HR (95% CI)	p-value
Low	Reference							
Middle	1.58 (1.17, 2.13)	0.002	1.55 (1.15, 2.10)	0.004	1.55 (1.14, 2.12)	0.005	1.67 (1.19, 2.33)	0.003
High	2.12 (1.57, 2.86)	<0.001	1.93 (1.43, 2.61)	<0.001	1.90 (1.39, 2.60)	<0.001	1.55 (1.09, 2.19)	0.014

Model 1: adjusted for age, sex, ethnicity.

Model 2: adjusted for age, sex, ethnicity and BMI.

Model 3: adjusted for age, sex, ethnicity, BMI, SBP, HbA1c, total cholesterol, LDL, non-HDL, eGFR, uACR, pre-existing CAD or PVD, ACE-inhibitor or ARB use.

We investigated whether sex, age, baseline eGFR and pre-existing CAD or PVD modified the association between NLR and outcomes. The interaction terms were not significant for any of the outcomes (all-cause mortality, MAVE and renal).

## Discussion

In this cohort of 959 individuals with T2DM, elevated NLR was associated with adverse outcomes including all-cause mortality, composite vascular complications, MAVE and progression of diabetic nephropathy. Interaction tests did not reveal any significant associations between sex and ethnicity, suggesting the generalizability of NLR as a predictor of all-cause mortality and adverse outcomes in a T2DM cohort in a Southeast Asian population.

The association between NLR with all-cause mortality, cardiovascular disease and progression of DKD are consistent with other populations. In the NHANES cohort comprising 3251 T2DM participants over a median follow-up period of 91 months, elevated NLR above 3.48 was associated with a 2-fold increase in all-cause mortality (11). In the post–hoc analysis of the SUSTAIN 6 and PIONEER randomized controlled trials involving over 6300 individuals with T2DM, there was a strong association between NLR and incident cardiovascular events, with patients in the highest NLR tertile (> 2.65) exhibiting a significant 2.1-fold increased risk of first MACE (17).

With respect to DKD, the associations between NLR with progression of renal impairment and albuminuria progression had been reported in other populations. A cross-sectional study of 4813 Chinese patients with T2DM reported the association between NLR with diabetic nephropathy (18). Compared to the lowest quartile of NLR, the odds of having diabetic nephropathy was increased by 2.5 fold with the highest NLR quartile (17). In the NHANES cohort involving more than 7100 participants with T2DM, elevated NLR was significantly associated with a 2.9-fold risk of diabetic nephropathy (8). The association between NLR and albuminuria was also demonstrated in three separate cohorts of patients in Egypt (19), Japan (20) and Turkey (21). Moreover, NLR has been studied for its role as a diagnostic marker of diabetic nephropathy. In a meta-analysis involving new patients with early diabetic nephropathy patients and more than 4800 individuals with diabetic nephropathy, NLR was shown to have a diagnostic sensitivity of 0.83 for early diabetic nephropathy and 0.73 for diabetic nephropathy (22).

The NLR reflects inflammation and immune dysregulation (8). Neutrophils are part of the innate immune system and serve as the first line of defense against external invasion such as bacterial infections (23). Apart from pathogens, several other types of molecules can activate innate immunity and promote neutrophils recruitment. These are known as damage associated molecular patterns (DAMPs), which bind to the corresponding pattern recognition receptors to induce downstream inflammatory processes (24). In T2DM, excessive glucose, free fatty acids and oxidized LDL are DAMPs that can activate neutrophils release and recruitment (25). On the other hand, lymphocytes are a crucial component of adaptive immunity (26). In addition to alterations in absolute lymphocyte quantity, studies have demonstrated qualitative changes in T lymphocytes subsets in T2DM. Profiling of the immune subsets of more than 15,000 individuals across the glycemic spectrum revealed a gradual increase in monocytes and granulocytes in T2DM (27). Other studies have found that hyperglycemia could be negatively correlated with certain lymphocyte subsets (28, 29). In particular, the reduction in CD4+ T cells could impair immune tolerance and exacerbate inflammatory responses, leading to the low-grade inflammation characteristic in T2DM (30). These dynamic changes in leukocyte profiles may contribute to the elevated NLR observed in T2DM (31).

Neutrophils play a pivotal role in the pathogenesis of atherosclerotic macroangiopathies and are involved in various stages of the process (32). The early stage of atherosclerosis is characterized by endothelial dysfunction, partly contributed by neutrophil adhesion to endothelial cells. Subsequent neutrophil migration into the subendothelial space is mediated by endothelial adhesion molecules that are upregulated by inflammatory cytokines such as tumour necrosis factor alpha (TNF-α) and interleukin (IL)-1β (25). In addition, crosstalk between neutrophils and other leukocyte subsets not only promotes the recruitment of monocytes to the endothelium but also induces macrophage polarization into the proinflammatory M1 phenotype (33), accelerating the formation of foam cells and atherosclerotic plaque development (34).

Lymphopenia reflects impaired adaptive immunity and is associated with atherosclerotic cardiovascular disease (35). In a large observational study involving more than 15,000 patients who underwent coronary angiography, lymphopenia was associated with a 1.3-fold increased risk for mortality (36). There are several mechanisms that could explain the association between lymphopenia and adverse cardiovascular outcomes. Firstly, activation of the adaptive immune system during a cardiovascular event could increase lymphocyte apoptosis (36). Secondly, lymphopenia could be a physiological response to stress, release of cortisol and catecholamines (9). Thirdly, acute stress redistributes circulating lymphocytes into the lymphoid organs (37) and a reduction in the lymphocytes pool may suggest depletion of the protective anti-inflammatory T regulatory cells (38), although further studies performing detailed subtyping of the subsets are required to draw conclusions about the contributory role of lymphocyte redistribution and cardiovascular disease.

The link between NLR and DKD reflects the crucial role of chronic inflammation (8). Elevated neutrophils in DKD could drive the synthesis of inflammasome NLRP3, stimulate the release of inflammatory cytokines, which promote renal fibrosis, glomerular and tubular damage (39). In addition, the distribution of leukocyte fractions had been studied in a cohort of T2DM patients with DKD confirmed by renal biopsy. Specifically, lower lymphocyte fraction, higher neutrophil fraction and high NLR were associated with reduced endothelial fenestrations respectively (40). Loss of endothelial fenestrations reduces glomerular permeability, increases structural resistance and can lead to the characteristic reduction in glomerular filtration seen in DKD progression (41).

There are several advantages in using NLR as a potential biomarker to predict adverse vascular complications and mortality in T2DM individuals. NLR is an easily accessible and reliable marker that can be performed routinely in the outpatient settings. In addition, the NLR provides insights about inflammation that may not be reflected by other biomarkers such as high-sensitivity C-reactive protein (hsCRP). A prospective observational study found discordant associations between NLR, hsCRP and cardiovascular outcomes over a median duration of 36 months of follow up. The risk of incident MACE was significantly higher in people with low hsCRP but high NLR (42). Another study involving more than 10,000 person years in 1,239 patients with type 2 diabetes, higher NLR was independently associated with vascular events independent of hsCRP (43). In a large pooled analysis of 5 randomized controlled trials with NLR data available from more than 60,000 participants, NLR outperformed absolute neutrophils count in predicting MACE, suggesting NLR reflecting residual inflammatory risks beyond hsCRP (10). Furthermore, this pooled analysis reported stability of the NLR in participants who received placebos (10). These evidence collectively support the utility of NLR as a potential clinical biomarker that not only informs future risks of cardiovascular complications at baseline, longitudinally overtime and also as a potential therapeutic surrogate marker of response especially if anti-inflammatory therapies are initiated.

### Strengths and limitations

We provide a comprehensive overview of the associations between NLR with all-cause mortality and vascular complications in a multiethnic cohort in a Southeast Asian population. The lack of interactions between ethnicity, baseline renal function, pre-existing CAD or PVD with NLR offers another layer of generalizability of NLR in diverse populations and supports the use of NLR as a potential stratification tool regardless of baseline renal function or pre-existing vascular disease. In addition, the participants were followed over a median duration of 9.4 years, providing insights of the impact of NLR on long-term outcomes. However, there are several limitations that need to be addressed. First, given the observational nature of this study, confounding associations between other risk factors could not be completely eliminated. Secondly, the nature of the data collection method based on real-world clinical documentation limited comprehensive tabulation of smoking status, physical activity and dietary intake, which could influence the association between NLR and MAVE. Thirdly, due to limited linkage of medical records across the nation, we were unable to fully ascertain all causes of mortality and there could be MAVE and composite renal outcomes that were not captured. Fourthly, we did not assess other microvascular complications including diabetic retinopathy and neuropathy.

### Conclusions and future directions

The NLR is a potentially cost effective and readily accessible clinical biomarker that may provide additional insights into the inflammatory state of individuals with T2DM. A routine NLR screening in the outpatient setting may provide further information about the long-term risks of vascular complications in T2DM. The trajectory of change in response to interventions, especially to anti-inflammatory agents, may expand its role as a surrogate marker of therapeutic response. Future clinical studies and randomized controlled trials should test the cost-effectiveness and long-term impact of implementing and routinely tracking NLR changes as a clinical biomarker with dynamic predictive value.

## Data Availability

The raw data supporting the conclusions of this article will be made available by the authors, without undue reservation, upon reasonable request.
